# Comparison of different modes of antibiotic delivery on gut microbiota depletion efficiency and body composition in mouse

**DOI:** 10.1186/s12866-020-02018-9

**Published:** 2020-11-11

**Authors:** Pauline Tirelle, Jonathan Breton, Gaëtan Riou, Pierre Déchelotte, Moïse Coëffier, David Ribet

**Affiliations:** 1 UNIROUEN, INSERM UMR 1073, Nutrition, Inflammation et dysfonction de l’axe intestin-cerveau, Normandie University, Rouen, France; 2 UNIROUEN, Institute for Research and Innovation in Biomedicine (IRIB), Normandie University, Rouen, France; 3 Nutrition Department, Rouen University Hospital, Rouen, France; 4 UNIROUEN, INSERM UMR 1234, PANTHER, Flow cytometry facility, Normandie University, Rouen, France; 5 INSERM UMR1073, Université de Rouen, UFR Santé - 22 Boulevard Gambetta, 76183 Rouen Cedex, France

**Keywords:** Gut microbiota, Gut microbiome, Intestinal microbiota, Intestinal microbiome, Antibiotics, Mouse model, Dysbiosis, Fecal bacteria, Antifungals, Escherichia

## Abstract

**Background:**

The use of animal models with depleted intestinal microbiota has recently increased thanks to the huge interest in the potential role of these micro-organisms in human health. In particular, depletion of gut bacteria using antibiotics has recently become popular as it represents a low cost and easy alternative to germ-free animals. Various regimens of antibiotics are used in the literature, which differ in composition, dose, length of treatment and mode of administration. In order to help investigators in choosing the most appropriate protocol for their studies, we compared here three modes of antibiotic delivery to deplete gut bacteria in C57Bl/6 mice. We delivered one of the most frequently used combination of antibiotics (a mix of ampicillin, neomycin, metronidazole and vancomycin) either ad libitum in drinking water or by oral gavage once or twice per day.

**Results:**

We quantified the global bacterial density, as well as the abundance of specific bacterial and fungal taxa, in mouse feces in response to antibiotics exposure. We observed that oral gavage once a day with antibiotics is not a reliable method as it occasionally triggers hyperproliferation of bacteria belonging to the Escherichia/Shigella taxon and leads, as a consequence, to a moderate decrease in fecal bacterial density. Antibiotics delivery by oral gavage twice a day or in drinking water induces in contrast a robust and consistent depletion of mouse fecal bacteria, as soon as 4 days of treatment, and is associated with an increase in fecal moisture content. Extending exposure to antibiotics beyond 7 days does not improve total bacteria depletion efficiency and promotes fungal overgrowth. We show in addition that all tested protocols impact neither gut microbiota recolonization efficiency, 1 or 2 weeks after the stop of antibiotics, nor mice body composition after 1 week of treatment.

**Conclusions:**

Our study provides key experimental data and highlights important parameters to consider before selecting an appropriate protocol for antibiotic-mediated depletion of gut bacteria, in order to optimize the accuracy and the reproducibility of results and to facilitate comparison between studies.

**Supplementary Information:**

**Supplementary information** accompanies this paper at 10.1186/s12866-020-02018-9.

## Background

The intestinal tract of mammals harbors trillions of micro-organisms which, altogether, constitute a complex and dynamic ecosystem. This ecosystem includes bacteria, archaea, fungi, protozoans and viruses that play key roles in the host physiology. Bacteria constitute an essential part of the gut microbiota. In humans, the intestine was estimated to contain about ~ 4.0 × 10^13^ bacteria per individual [1]. The dominant intestinal bacterial phyla present in humans are Firmicutes (~ 60–65%), Bacteroidetes (~ 20–25%), Proteobacteria (~ 5–10%) and Actinobacteria (~ 3%) [2]. The role of the intestinal microbiota in human physiology has gained a huge interest during the last 10 years. Alteration in the composition or metabolic activities of the gut microbiota has been linked to diseases such as chronic inflammatory diseases, obesity, cardiovascular diseases, cancers, or even behavioral disorders [3–6]. However, most studies published so far are only observational and show associations between gut microbiota and diseases, without proving causation [3]. Manipulation of gut microbiota in animal models constitutes a key experimental approach to demonstrate causality between gut microbiota dysbiosis and the occurrence of symptoms distinctive of a given disease. In particular, mouse models with depleted gut microbiota have been increasingly used over the last years to get further insights into the role of these micro-organisms in the occurrence or chronicity of human diseases.

Two main methods are generally used to obtain mice depleted for intestinal bacteria: germ-free mice and antibiotic-treated mice. Germ-free mice are considered as the gold standard to study the effect of the complete absence of microbes, to establish mice with precisely defined microbiota composition (gnotobiotic mice) or to perform intestinal microbiota transfer experiments [7–9]. However, this model remains inaccessible to many investigators as it requires specialized facilities (these animals being bred in isolators to keep them free of detectable bacteria, eukaryotes and viruses) and are very costly. As a consequence, treatment of animals with broad-spectrum antibiotics has quickly emerged as a low cost and easy alternative method to deplete mice gut bacteria. Various regimens of antibiotics have been used in the literature, which differ in composition, dose, length of treatment and mode of administration [8]. In order to help investigators to select the most appropriate protocol for their research project, we compared here three modes of administration (ad libitum in drinking water or via oral gavage once or twice per day) of one of the most frequently used combination of antibiotics (a mix of ampicillin, neomycin, metronidazole and vancomycin broadly targeting gram-positive, gram-negative and anaerobic bacteria) [8, 10–19]. We quantified the efficiency of bacteria depletion in mouse feces induced by these different modes of administration, the impact of these antibiotics on the abundance of several bacterial and fungal taxa and the speed of gut bacteria recolonization after the stop of antibiotics. We also monitored the impact of antibiotics on fecal moisture content and on mouse body composition (lean and fat mass).

## Results

In order to compare how different modes of antibiotics delivery may affect mouse gut microbiota and body composition, we compared 4 groups of animals: one group received antibiotics by oral gavage twice a day (2xG-ATB group), one group received antibiotics by oral gavage once a day (1xG-ATB group), one group had antibiotics added in drinking water (DW-ATB group) and the last group did not receive antibiotics (no ATB group). Animals receiving antibiotics were treated during 12 days and were then kept for another 14 days, without antibiotics.

**Fig. 1:**
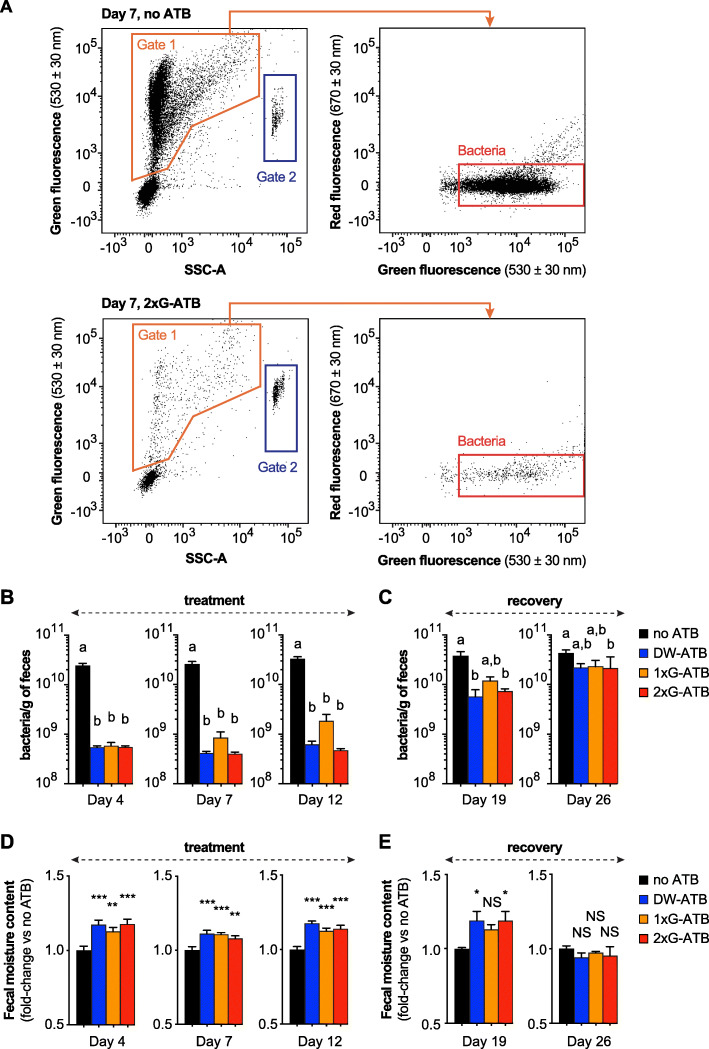
Effect of antibiotics on fecal bacterial density and fecal moisture content in mice. **a** Flow cytometry gating strategy for quantification of bacterial densities. Acquisition plots for fecal samples from untreated mice (no ATB; top) and mice treated with antibiotics by oral gavage twice a day during 1 week (2xG-ATB; bottom) are represented. A first gate was defined based on green fluorescence/SSC-A channels to exclude debris or background events (gate 1). A secondary gating was performed on events from gate 1 to count bacteria and to exclude events with low green fluorescence and high red auto-fluorescence intensities. The volume analyzed by the flow cytometer for each sample was determined by quantifying the number of calibrating beads detected in gate 2. **b** Flow cytometry-based quantification of bacterial density in mice feces during antibiotic treatment (means ± SEMs, *n* = 12–15; Kruskal-Wallis test with Dunn’s correction). **c** Flow cytometry-based quantification of bacterial density in mice feces during recovery from antibiotic treatment (means ± SEMs, *n* = 9–10; Kruskal-Wallis test with Dunn’s correction). **d** Moisture content in mice feces during antibiotic treatment (values are expressed as fold-change compared to untreated mice; means ± SEMs, *n* = 12–15; one-way ANOVA with Holm-Sidak’s correction). **e** Moisture content in mice feces during recovery from antibiotic treatment (values are expressed as fold-change compared to untreated mice; means ± SEMs, *n* = 8–10; one-way ANOVA with Holm-Sidak’s correction). Labeled plots without a common letter differ; *P* < 0.05; *, *P* < 0.05 versus “no ATB” group; **, *P* < 0.01; ***, *P* < 0.001; NS, not significant

To evaluate the efficiency of antibiotic-mediated gut bacteria depletion, we monitored the density of fecal bacteria by both flow cytometry (using a fluorescent dye staining nucleic acids; Fig. 1a) and by qPCR on DNA extracted from feces (using 16S rRNA specific primers; Table S1). To validate our flow cytometry-based quantifications, we compared the fecal bacterial loads estimated by either flow cytometry or qPCR, using eubacteria-specific primers, in a set of 54 fecal samples coming from both untreated and antibiotic-treated mice. Although these two methods present independent biases which hampers direct comparison [20, 21], we observed that the fecal densities determined by our two approaches were strongly correlated (Spearman’s r = 0.85, two-tailed *P* < 0.0001) (Figure S1).

We used our flow cytometry-based approach to monitor fecal bacterial density during the 12 days of antibiotic treatment (Fig. 1b) and during the 14 days of recovery, after the stop of antibiotics (Fig. 1c). We observed that all modes of antibiotic administration induce a strong decrease in the density of fecal bacteria in comparison to untreated mice (Fig. 1b). This decrease is detected after only 4 days of treatment. For mice receiving antibiotics by oral gavage twice per day or in drinking water, the decrease in fecal bacterial density was strong (> 20-fold decrease) and not significantly different between Day 4 and 12 (*P* > 0.1; Mixed-effects analysis with Tukey’s correction). This suggests that maintaining antibiotics administration more than 4 days does not further decrease fecal bacterial loads. In addition, we did not observe significant differences in the bacterial depletion efficiencies between these two modes of antibiotic delivery at Day 4, 7 and 12, indicating that these two protocols have similar impact on the global fecal bacterial loads. For mice receiving antibiotics by oral gavage once per day, we noticed strong variations in bacterial depletion efficiency between experiments. Among 4 independent experiments, we observed a strong decrease in fecal bacterial densities at Day 7 only in two experiments. For the other two experiments, we observed a limited decrease in fecal bacterial densities, which correlated with the hyperproliferation of specific Gammaproteobacteria (see below; Figure S2).

We then evaluated the efficiency of gut microbiota recolonization 1 or 2 weeks after the stop of antibiotic treatment. After 1 week of recovery (Day 19), we observed an increase in the density of fecal bacteria which illustrates partial gut recolonization (Fig. 1c). After 2 weeks (Day 26), all mice recovered at least 50% of their initial gut bacteria and we did not observe significant differences in fecal bacterial densities between the different groups of antibiotic-treated mice (Fig. 1c). These results suggest that the gut is quickly recolonized after the stop of antibiotics and that there is no major effect of the mode of antibiotic delivery on the gut recolonization efficiency in the tested conditions.

In parallel to the quantification of fecal bacterial density, we evaluated the moisture content in mice feces during the protocol. All mice treated with antibiotics exhibit a significant increase in fecal moisture content at Day 4, Day 7 and Day 12 (Fig. 1d). After 1 week of recovery, mice receiving antibiotics via drinking water or oral gavage twice a day still exhibit increased moisture content in feces, whereas no significant differences between control and antibiotic-treated mice were observed after 2 weeks (Fig. 1e). This progressive return to normal of fecal moisture content is consistent with the previously observed gut recolonization of mice by bacteria 2 weeks after the stop of antibiotics (Fig. 1c).

**Fig. 2:**
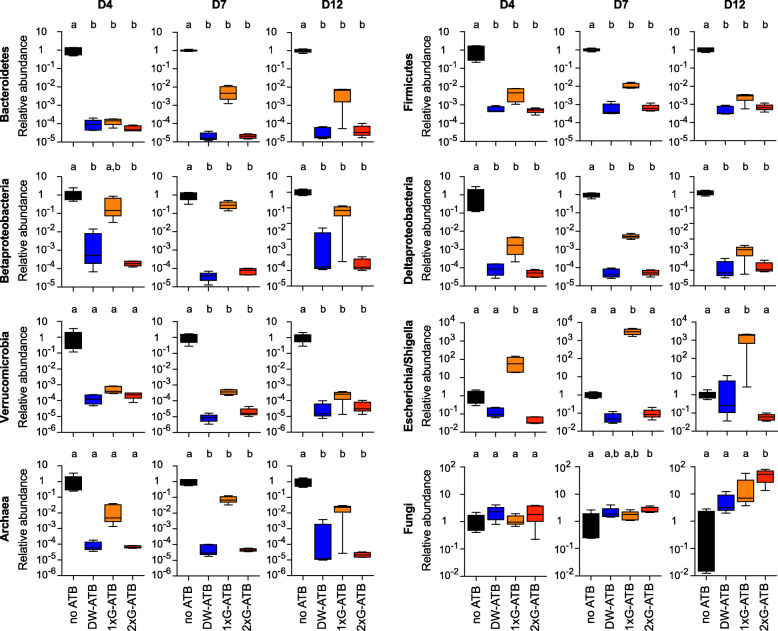
Fecal abundance of different bacterial and fungal taxa. Relative quantification of different bacterial and fungal taxa in mouse feces after 4, 7 or 12 days of treatment, as determined by qPCR analysis (values are expressed as fold-change of the mean abundance in untreated mice and represented as whisker plots with minimum and maximum values, *n* = 4–5 per group; Labeled plots without a common letter differ; *P* < 0.05, one-way ANOVA with Tukey’s correction)

**Fig. 3:**
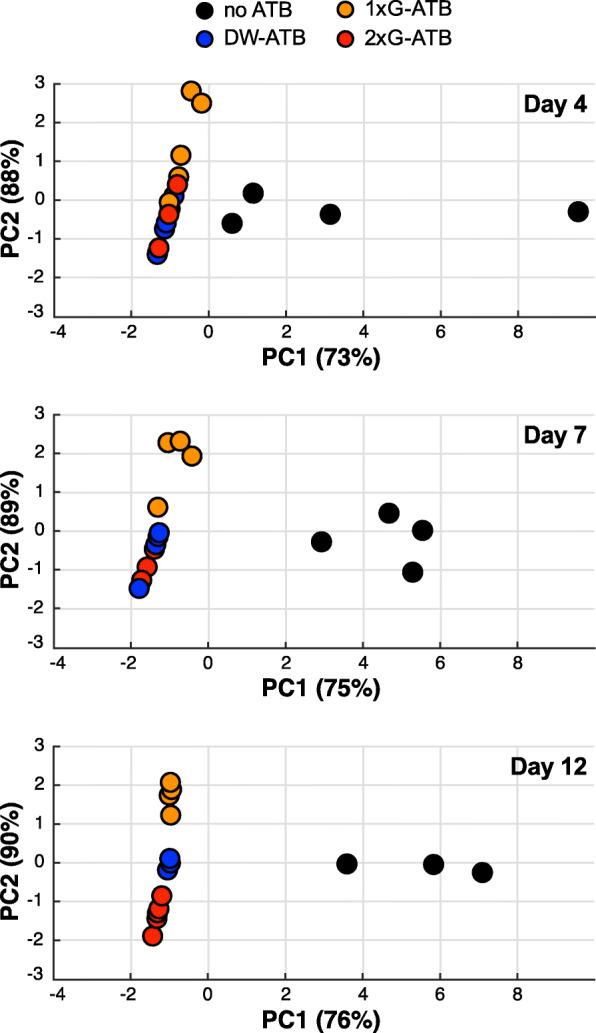
Effect of antibiotics on mouse gut microbiota composition. Principal components analyses of mice treated or not with antibiotics during 4, 7 or 12 days, based on the quantification of nine bacterial and fungal taxa by qPCR

In order to better characterize the impact of antibiotics on the composition of the gut microbiota, we monitored by qPCR the abundance of several bacterial taxa, including major phyla of mouse intestinal microbiota: Eubacteria, Archaea, Bacteroidetes, Firmicutes, Betaproteobacteria, Deltaproteobacteria, Escherichia/Shigella and Verrucomicrobia. We quantified the relative abundance of these taxa in mouse feces after 4, 7 and 12 days of antibiotic treatment (Fig. 2) and compared the deduced microbiota structures using principal components analyses (Fig. 3). These analyses were first performed on one of the two experiments described above showing a weak decrease in bacterial density after antibiotic delivery by one daily oral gavage. As expected, untreated mice clearly cluster separately from antibiotic-treated mice from Day 4 to Day 12. In agreement with our quantification of fecal bacterial densities, we observed that mice receiving antibiotics by oral gavage twice a day or in drinking water cluster together, whereas mice receiving antibiotics by oral gavage once per day constitute a separate group (Fig. 3 and Figure S2). The detailed analysis of the relative abundance of bacterial taxa in mice receiving antibiotics by twice daily oral gavages or in drinking water revealed a strong and persistent decrease for all tested taxa from Day 4 to Day 12 (Fig. 2). In contrast, we observed for mice treated once daily by oral gavage a weaker decrease in the abundance of several independent taxa and a strong hyperproliferation of the Escherichia/Shigella taxon (Fig. 2 and Figure S2). A similar analysis was then performed on the second experiment showing a weak decrease in bacterial density after antibiotics delivery by oral gavage once per day. It also revealed an hyperproliferation of the Escherichia/Shigella taxon in this group of mice (Figure S2), thereby confirming that this mode of delivery occasionally increases the abundance of specific Gammaproteobacteria.

We took advantage of these qPCR quantifications to evaluate the efficiency of Amphotericin-B, which was co-administrated to mice in our experimental setup to prevent fungal overgrowth, as a consequence of bacterial depletion. We quantified the abundance of fungi in mice feces using 18 s rRNA specific primers and probes (Fig. 2). We did not observe significant increases in fungi levels after 4, 7 and 12 days of treatment for mice receiving antibiotics in drinking water or by oral gavage once a day (an increase was observed for both treatments at Day 12, which did not reach significance; Fig. 2). For mice receiving antibiotics twice a day by oral gavage, a significant increase in fungal abundance was observed after 7 and 12 days of treatment (× 2.7 ± 0.7 and × 29.9 ± 15.2, respectively, compared to untreated mice). This increase is indicative of a fungal overgrowth in these particular conditions, despite the administration of Amphotericin-B (Fig. 2).

**Fig. 4:**
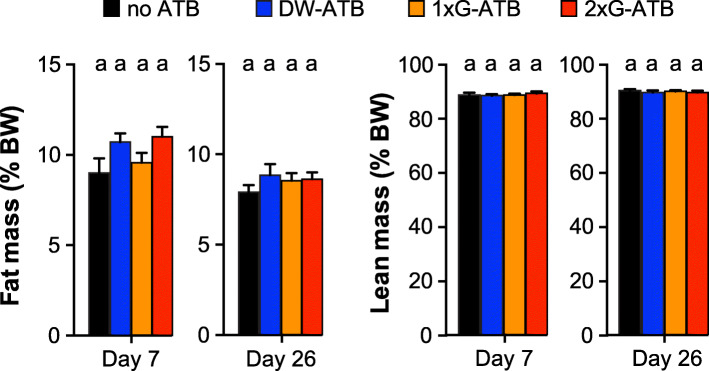
Effect of antibiotics on body composition in mice. Fat and lean masses at Day 7 and Day 26 (values are expressed as percentage of body weight; means ± SEMs, *n* = 9–10; Labeled means without a common letter differ, *P* < 0.05; Kruskal-Wallis test with Dunn’s correction)

We finally monitored the impact of antibiotic treatments on mice body weight and composition. No significant differences in body weight was observed between control mice and mice receiving antibiotics (either in drinking water or via oral gavage once per day) between Day 4 and Day 12. Mice receiving antibiotics by oral gavage twice per day showed a slight and transient decrease of body weight at Day 7 (< 5% decrease compared to untreated mice). In parallel, we evaluated mice body composition after 7 days of antibiotic treatment or at the end of the recovery period (Day 26). We did not observe any significant differences in the percentage of fat or lean mass between untreated and antibiotic-treated mice (Fig. 4). Antibiotics have thus no effect on mouse body composition after 1 week of antibiotic treatment or 2 weeks after the stop of antibiotics in the tested conditions.

## Discussion

Antibiotic-mediated depletion of gut bacteria in animals constitutes an interesting alternative to germ-free animals to study microbiota-dependent phenotypes. Indeed, experimentation on germ-free mice is associated with several technical difficulties including the breeding in isolators, the regular monitoring for contamination or the necessity to rederive each individual mouse strain to be studied under germ-free conditions. In addition, germ-free animals present several developmental defects and are impaired in the early education of the immune system [22]. Antibiotic-mediated depletion of gut bacteria is, in contrast, inexpensive, does not require specialized equipment, can be applicable to any genotype, and does not present developmental defects. However, this approach has also limitations including the incomplete depletion of gut bacteria compared to germ-free animals, a possible effect of antibiotics on eukaryotic cells and the diversity of antibiotic regimens used so far between published studies that challenges comparison of the observed phenotypes [8].

In order to help investigators selecting the most appropriate protocol for their studies, we provide here experimental data on the differential impact of three modes of antibiotic administration. Of note, in our experimental setup, mice were co-housed in a treatment-dependent manner. This co-exposure of mice to each other during antibiotics treatment and after recovery may affect the composition of their gut microbiota and results may differ if mice were singly-housed. Our data show that delivering antibiotics by oral gavage only once per day results in variable fecal bacteria depletion efficiencies. Indeed, this mode of delivery may occasionally lead to the hyperproliferation of Gammaproteobacteria, which partially compensates the decrease in the abundance of other taxa and leads to a completely dysbiotic microbiota. This mode of antibiotic delivery thus seems inappropriate. In contrast to this first mode of administration, antibiotic delivery in drinking water or via oral gavage twice per day induces a strong and consistent depletion of fecal bacteria (> 20-fold decrease after 4 days of treatment). The observed efficiencies of bacteria depletion with these two protocols are of the same order of magnitude than the ones reported in other studies using similar protocols [8, 11–13, 15, 17, 19]. Interestingly, no significant difference in fecal bacteria depletion was observed between these protocols. Although the amount of ingested antibiotics is less controlled for mice receiving antibiotics via drinking water (as it depends on the daily water intake, which varies between animals), our result shows that it does not significantly change gut bacteria depletion efficiency compared to two daily oral gavages. Interestingly, we did not observe significant improvement in fecal bacteria depletion between Day 4 and Day 12 for these protocols. In contrast, we observed an increase in fungi abundance between Day 7 and 12. This suggests that extending antibiotic treatment beyond 7 days may not be justified as it increases the amount of antibiotics used, the total duration of animal experimentations and the risk of fungal overgrowth. We further show in this study that all tested modes of antibiotic administration do not impact mice body composition after 7 days of treatment, nor gut microbiota recolonization efficiency after the removal of antibiotics. We also observed that all protocols induce an increase in fecal moisture content between Day 4 and Day 12, that is restored 2 weeks after the stop of antibiotics. This increase in fecal moisture content is consistent with previous reports [23, 24]. As intestinal transit time was shown to be increased by antibiotic treatment in several studies [23–25], the observed increase in fecal moisture content is probably linked to alterations in intestinal fluid secretions as a consequence of antibiotic-mediated bacteria depletion [26–28].

Our results suggest that antibiotics provided in drinking water or via oral gavage twice per day constitute equivalent protocols (for treatment periods shorter than 7 days). Other parameters should thus be considered by investigators before selecting one of these two protocols. It has been reported that administration of broad-spectrum antibiotics in drinking water may increase baseline morbidity and mortality in mice [12, 13]. In particular, the taste of some antibiotics may refrain mice from drinking the antibiotic-containing solution, leading to dehydration and significant loss in body weight (> 20% loss after 3–6 days of treatment) [12, 13]. In our protocol, we did not observe any significant changes in body weight for mice drinking antibiotic-containing water, even after 12 days of treatment. This divergence of results is probably due to either differences in the strains/genotypes of the animals used or to differences in the housing environment. Thus, a preliminary study assessing the potential dislike of a given mouse model to antibiotics in drinking water should be performed by investigators before going on with larger studies using this protocol. The addition of sweeteners in drinking water might also be an option to mask any potential antibiotic-associated bitterness [18, 29, 30].

The choice of the mode of antibiotic administration should also take into account the type of physiological parameters and phenotypes that will be evaluated during animal experiments. Animal behavior monitoring or measuring of parameters strongly affected by animal stress, such as intestinal permeability for example, might be difficult to assess if antibiotics are provided by daily gavages. Indeed, this procedure may strongly modify the basal level of animal stress (by either increasing it due to the repeated handling of animals or, in contrast, decreasing it via a “nursing” effect) and thus confounds the interpretation of results. Administration of antibiotics via drinking water for these particular parameters/phenotypes would then constitute the best alternative to limit interventions on animals.

## Conclusions

Considering the current huge interest for elucidating the role of the gut microbiota in human health and disease, the use of microbiota-depleted animal models will become more and more frequent. Selecting the most appropriate depletion protocol will be critical for these future studies to ensure the accuracy, reproducibility and the translation of the obtained results to humans.

## Methods

### Animals

Eight-weeks-old C57Bl/6JRj male mice (Janvier Labs, Le-Genest-Saint-Isle, France) were housed at 23 °C (5 animals/cage) with a 12-h light-dark cycle (light phase from 7:00 am to 7:00 pm) in regular open cages. All animals were fed with a non-sterilized standard rodent diet (3430.PM.S10, Serlab, France). Drinking water was not sterilized. After 1 week of acclimatization to the animal facility, animals were split in four groups (5 animals/group): one group had no antibiotic treatment and were gavaged twice a day with drinking water (no ATB group), one group received antibiotics by oral gavage twice a day at 10:00 am and 5:00 pm (2xG-ATB group), one group received antibiotics by oral gavage once a day at 10:00 am (1xG-ATB group) and one group received antibiotics directly added in drinking water (DW-ATB group). For antibiotics added directly in drinking water, the final concentrations used were: 0.01 mg/mL Amphotericin-B (Sigma-aldrich), 1 mg/mL Ampicillin (Sigma-aldrich), 1 mg/mL Neomycin trisulfate salt hydrate (Sigma-aldrich), 1 mg/mL Metronidazole and 0.5 mg/mL Vancomycin hydrochloride (Sigma-aldrich) (these concentrations were based on the estimation of a daily drunk volume of 5 mL per animal) [10]. Antibiotic-containing drinking water was renewed every day. For oral gavages, mice received a volume of 10 μL/g body weight of drinking water supplemented with 0.1 mg/mL Amphotericin-B, 10 mg/mL Ampicillin, 10 mg/mL Neomycin trisulfate salt hydrate, 10 mg/mL Metronidazole and 5 mg/mL Vancomycin hydrochloride [13]. This solution was delivered with a stainless steel tube without prior sedation of the mice. To prevent fungal overgrowth in the antibiotic-treated animals, mice were pre-treated with Amphotericin-B for 3 days before the beginning of the protocol [13]. As for antibiotic treatment, Amphotericin-B was either directly added in drinking water (final concentration of 0.01 mg/mL) for the DW-ATB group, or delivered by oral gavage for 1xG-ATB and 2xG-ATB groups (10 μL/g body weight of drinking water supplemented with 0.1 mg/mL Amphotericin-B) [13]. Four independent animal series were performed. At the end of the study, all animals were euthanized by an intraperitoneal injection of an overdose of ketamine (200 mg/kg BW) and xylazine (20 mg/kg BW). Cessation of heartbeats and non-responsiveness to noxious stimulus (hind paw pinch) were used as criteria to verify death.

### Quantification of fecal bacterial density by flow cytometry

Fecal samples were weighted, resuspended in 1X PBS (in order to obtain 2% weight/volume fecal suspensions) and mechanically homogenized by a bead-beating step (5 min of beating after addition of 2 glass beads per sample). Debris from fecal suspensions were removed by centrifugation (20 s at 300×*g* at room temperature). Clarified fecal suspensions were further diluted 200 times in 1X PBS, stained for 15 min with Syto™ BC (1:2000 dilution; Molecular Probes) and fixed overnight in 1X PBS-0.5% PFA. In parallel, an overnight culture of *E. coli* grown in LB was prepared. Bacteria were centrifuged at 18,000×*g* for 5 min at room temperature and resuspended in 1X PBS. Several dilutions of *E. coli* were prepared (5.10^6^ to 2.10^4^ bacteria/mL) and stained for 15 min with Syto™ BC (1:2000 dilution). *E. coli* were then fixed overnight in 1X PBS-0.5% PFA. Bacterial density of the initial *E. coli* culture was determined in parallel by plating and quantifying colony-forming units. These suspensions of stained *E. coli* were used in each experiment to validate the efficiency of bacterial detection and the linearity of bacterial numeration. Calibrating beads were added to fecal and *E. coli* samples to allow for absolute quantification by flow cytometry (polystyrene microspheres; Bacteria Counting Kit, Molecular Probes).

Microbial cells numeration was performed using a LSRFortessa™ flow cytometer (Becton Dickinson), according to previously published methods [21, 31]. Excitations were performed at 488 and 561 nm, while fluorescence intensities were collected at 530 ± 30 nm and 670 ± 30 nm respectively. The FlowJo software (TreeStar Inc.) was used to gate and separate the microbial fluorescence events from the fecal sample background. Non-stained fecal samples and 1x PBS solution were used to define the gates. Instrument settings and gating strategy were kept identical for all samples.

### Quantification of fecal microorganisms by quantitative PCR

Quantitative real-time polymerase chain reaction (qPCR) was performed on DNA samples extracted from mice feces to monitor the level of several bacterial and fungal taxa, as described previously [32, 33]. In brief, DNA from mice feces were extracted using the QIAamp DNA Stool Mini Kit (QIAGEN), including a bead-beating step (0.1 mm zirconia silica beads, BioSpec products, Bartlesville, USA) [34]. To quantify bacterial taxa, qPCR were performed using Itaq Universal SYBR Green Supermix (BioRad) with 16S rRNA specific primers. To quantify fungi, qPCR were performed using Itaq Universal Probes Supermix (BioRad) with 18S rRNA specific primers and a FAM-labeled probe. Primer and probe sequences are detailed in Table S1. Serial dilutions of fecal DNA were included on each plate to generate a relative curve and to integrate primer efficiency in the calculations. For the detection of total Eubacteria, the Cq of each sample were compared with a standard curve made by diluting genomic DNA extracted from a pure culture of *E. coli,* for which cell counts were determined by plating and quantifying colony-forming units.

### Quantification of fecal moisture content

Fecal moisture content was determined as the percentage of fecal mass loss after lyophilization (48 h at 37 °C).

### Whole body composition

Whole body composition was assessed on vigil animals at Day 7 and Day 26 using nuclear magnetic resonance (EchoMRI EMR-185, Houston, Texas, USA).

### Statistical analysis

Comparison of fecal bacterial density between groups at different time points during the protocol and lean and fat masses between groups at Day 7 and 26 were performed using Kruskal-Wallis test with Dunn’s correction. Comparison of bacterial and fungal taxa levels quantified by qPCR were performed using one-way ANOVA with Tukey’s correction. Comparison of fecal moisture content between groups at different time points during the protocol were performed using one-way ANOVA with Holm-Sidak’s correction. Statistical analyses were performed with GraphPad Prism 6 (GraphPad Software, San Diego, USA). Principal components analyses were computed with MATLAB (version R2018a, The Mathworks, Inc.; Natick, USA).

## Supplementary Information

**Additional file 1: Figure S1.** Comparison between flow cytometry and qPCR-based quantifications of fecal bacterial densities. Analysis by both flow cytometry and qPCR-based methods of 54 fecal samples collected from mice treated or not with antibiotics during 4, 7 or 12 days. The bacterial densities measured by these two methods are strongly correlated (Spearman’s correlation coefficient *r* = 0.85; two-tailed *P* < 0.0001).

**Additional file 2: Figure S2.** Hyperproliferation of the Escherichia/Shigella taxon in mice receiving antibiotics by oral gavage once per day. A. Principal components analyses at Day 7 of mice treated or not with antibiotics, based on the quantification of nine bacterial and fungal taxa by qPCR. Two independent experiments are represented, which both exhibit a weak decrease in fecal bacterial density at Day 7 for mice receiving antibiotics by oral gavage once per day. B, Relative quantification by qPCR of the Eubacteria and Escherichia/Shigella taxa in mouse feces at Day 7 of the experiments shown in (A) (values are expressed as fold-change of the mean abundance in untreated mice and represented as whisker plots with minimum and maximum values, *n* = 4–5 per group; Labeled plots without a common letter differ; *P* < 0.05, one-way ANOVA with Tukey’s correction). In both experiments, the weak decrease in fecal bacterial density for mice receiving antibiotics by oral gavage once per day is correlated with an hyperproliferation of the Escherichia/Shigella taxon.

**Additional file 3 :Table S1.** Primer and probe sequences.

## Data Availability

All data generated or analyzed during this study are included in this published article.

## Abbreviations

ATB: antibiotics
DW: Drinking water
1xG/2xG: 1 gavage per day/2 gavages per day
PBS: Phosphate Buffered Saline
PFA: Paraformaldehyde
LB: Luria broth
