# Supplementary material for: Comparison of different modes of antibiotic delivery on gut microbiota depletion efficiency and body composition in mouse
Source: BMC Microbiol. 2020 Nov 11;20:340. doi: 10.1186/s12866-020-02018-9 (PMC7657353; doi:10.1186/s12866-020-02018-9)
Supplement: Supplementary file 1 — Additional file 1: Figure S1. Comparison between flow cytometry and qPCR-based quantifications of fecal bacterial densities. Analysis by both flow cytometry and qPCR-based methods of 54 fecal samples collected from mice treated or not with antibiotics during 4, 7 or 12 days. The bacterial densities measured by these two methods are strongly correlated (Spearman’s correlation coefficient r = 0.85; two-tailed P < 0.0001). [file 12866_2020_2018_MOESM1_ESM.pdf]

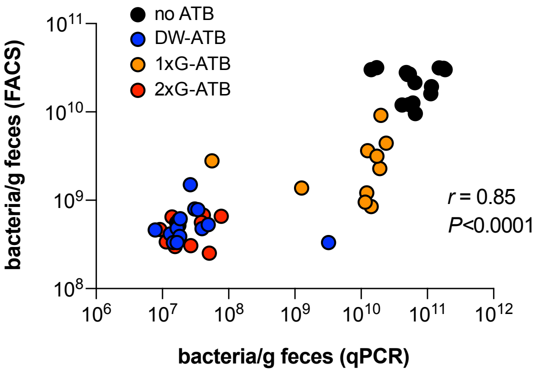

Figure S1: Comparison between flow cytometry and qPCR-based quantifications of fecal bacterial densities.

Analysis by both flow cytometry and qPCR-based methods of 54 fecal samples collected from mice treated or not with antibiotics during 4, 7 or 12 days. The bacterial densities measured by these two methods are strongly correlated (Spearman's correlation coefficient r=0.85; two-tailed P<0.0001).
