# Supplementary material for: Comparison of different modes of antibiotic delivery on gut microbiota depletion efficiency and body composition in mouse
Source: BMC Microbiol. 2020 Nov 11;20:340. doi: 10.1186/s12866-020-02018-9 (PMC7657353; doi:10.1186/s12866-020-02018-9)
Supplement: Supplementary file 2 — Additional file 2: Figure S2. Hyperproliferation of the Escherichia/Shigella taxon in mice receiving antibiotics by oral gavage once per day. A. Principal components analyses at Day 7 of mice treated or not with antibiotics, based on the quantification of nine bacterial and fungal taxa by qPCR. Two independent experiments are represented, which both exhibit a weak decrease in fecal bacterial density at Day 7 for mice receiving antibiotics by oral gavage once per day. B, Relative quantification by qPCR of the Eubacteria and Escherichia/Shigella taxa in mouse feces at Day 7 of the experiments shown in (A) (values are expressed as fold-change of the mean abundance in untreated mice and represented as whisker plots with minimum and maximum values, n = 4–5 per group; Labeled plots without a common letter differ; P < 0.05, one-way ANOVA with Tukey’s correction). In both experiments, the weak decrease in fecal bacterial density for mice receiving antibiotics by oral gavage once per day is correlated with an hyperproliferation of the Escherichia/Shigella taxon. [file 12866_2020_2018_MOESM2_ESM.pdf]

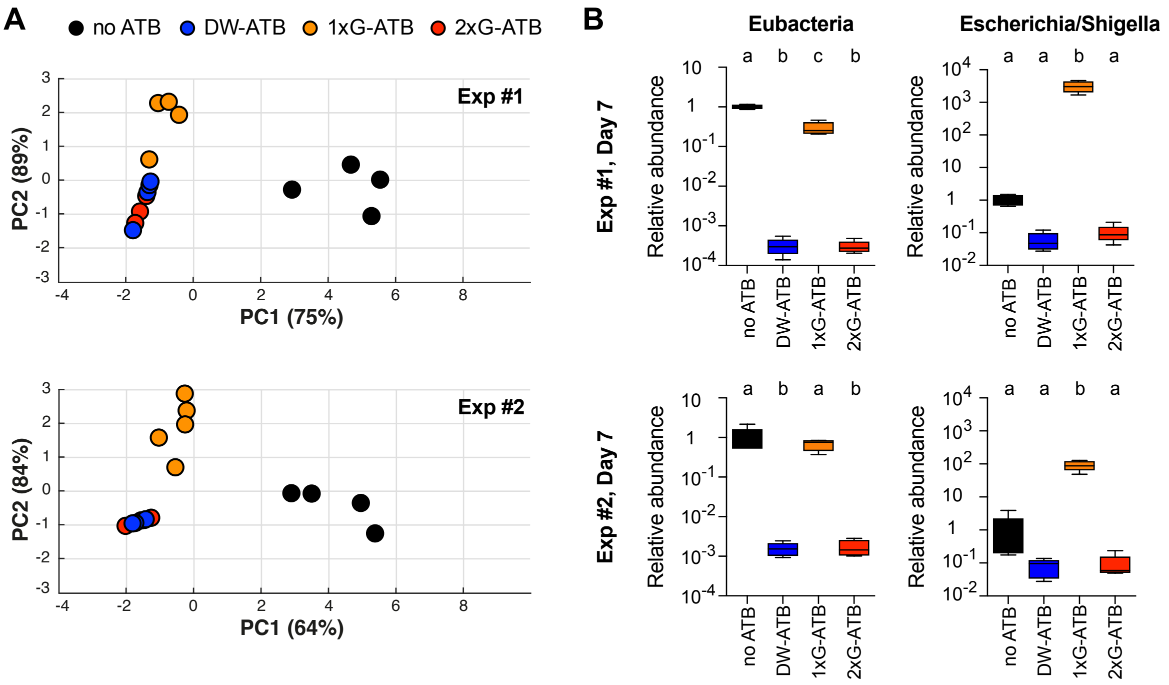

Figure S2: Hyperproliferation of the Escherichia/Shigella taxon in mice receiving antibiotics by oral gavage once per day.

A. Principal components analyses at Day 7 of mice treated or not with antibiotics, based on the quantification of nine bacterial and fungal taxa by qPCR. Two independent experiments are represented, which both exhibit a weak decrease in fecal bacterial density at Day 7 for mice receiving antibiotics by oral gavage once per day. B, Relative quantification by qPCR of the Eubacteria and Escherichia/Shigella taxa in mouse feces at Day 7 of the experiments shown in (A) (values are expressed as fold-change of the mean abundance in untreated mice and represented as whisker plots with minimum and maximum values, *n*=4-5 per group; Labeled plots without a common letter differ; *P*<0.05, one-way ANOVA with Tukey's correction). In both experiments, the weak decrease in fecal bacterial density for mice receiving antibiotics by oral gavage once per day is correlated with an hyperproliferation of the Escherichia/Shigella taxon.
