# Supplementary material for: Comparison of different modes of antibiotic delivery on gut microbiota depletion efficiency and body composition in mouse
Source: BMC Microbiol. 2020 Nov 11;20:340. doi: 10.1186/s12866-020-02018-9 (PMC7657353; doi:10.1186/s12866-020-02018-9)
Supplement: Supplementary file 3 — Additional file 3 :Table S1. Primer and probe sequences. [file 12866_2020_2018_MOESM3_ESM.docx]

**Supplemental Table 1: Primer and probe sequences**

| Taxon | Forward | Reverse | T an^a^ | Supplemental  reference |
| --- | --- | --- | --- | --- |
| *Archaea* | GYGCASCAGKCGMGAAW | TTACCGCGGCKGCTG | 48.5 | [1] |
| *Eubacteria* | ACTCCTACGGGAGGCAGCAG | ATTACCGCGGCTGCTGG | 60 | [2] |
| *Firmicutes* | ATGTGGTTTAATTCGAAGCA | AGCTGACGACAACCATGCAC | 51 | [3] |
| *Bacteroidetes* | CATGTGGTTTAATTCGATGAT | AGCTGACGACAACCATGCAG | 51 | [3] |
| *Betaproteobacteria* | GGGGAATTTTGGACAATGGG | ACGCATTTCACTGCTACACG | 58 | [4] |
| *Deltaproteobacteria* | GGTGTAGGAGTGAARTCCGT | TACGTGTGTAGCCCTRGRC | 62 | [5] |
| *Escherichia/Shigella* | CATTGACGTTACCCGCAGAAGAAG | CTCTACGAGACTCAAGCTTGC | 62 | [6] |
| *Verrucomicrobia* | GAATTCTCGGTGTAGCA | GGCATTGTAGTACGTGTGCA | 59 | [5] |
| *Fungi* | GGRAAACTCACCAGGTCCAG | GSWCTATCCCCAKCACGA | 56 | [7] |
|  | + probe: TGGTGCATGGCCGTT | |  |  |

^a^ Temperature of annealing

**Supplemental references**

1. Klindworth A, Pruesse E, Schweer T, Peplies J, Quast C, Horn M, et al. Evaluation of general 16S ribosomal RNA gene PCR primers for classical and next-generation sequencing-based diversity studies. Nucleic Acids Res. 2013;41(1):e1; doi: 10.1093/nar/gks808.

2. Fierer N, Jackson JA, Vilgalys R, Jackson RB. Assessment of soil microbial community structure by use of taxon-specific quantitative PCR assays. Appl Environ Microbiol. 2005;71(7):4117-20; doi: 10.1128/AEM.71.7.4117-4120.2005.

3. Queipo-Ortuno MI, Seoane LM, Murri M, Pardo M, Gomez-Zumaquero JM, Cardona F, et al. Gut microbiota composition in male rat models under different nutritional status and physical activity and its association with serum leptin and ghrelin levels. PLoS One. 2013;8(5):e65465; doi: 10.1371/journal.pone.0065465.

4. Muhling M, Woolven-Allen J, Murrell JC, Joint I. Improved group-specific PCR primers for denaturing gradient gel electrophoresis analysis of the genetic diversity of complex microbial communities. ISME J. 2008;2(4):379-92; doi: 10.1038/ismej.2007.97.

5. Hermann-Bank ML, Skovgaard K, Stockmarr A, Larsen N, Molbak L. The Gut Microbiotassay: a high-throughput qPCR approach combinable with next generation sequencing to study gut microbial diversity. BMC Genomics. 2013;14:788; doi: 10.1186/1471-2164-14-788.

6. Bartosch S, Fite A, Macfarlane GT, McMurdo ME. Characterization of bacterial communities in feces from healthy elderly volunteers and hospitalized elderly patients by using real-time PCR and effects of antibiotic treatment on the fecal microbiota. Appl Environ Microbiol. 2004;70(6):3575-81; doi: 10.1128/AEM.70.6.3575-3581.2004.

7. Liu CM, Kachur S, Dwan MG, Abraham AG, Aziz M, Hsueh PR, et al. FungiQuant: a broad-coverage fungal quantitative real-time PCR assay. BMC Microbiol. 2012;12:255; doi: 10.1186/1471-2180-12-255.
